# Let us shape our future with *GTCS Cases: GTCC*!

**DOI:** 10.1186/s44215-022-00002-5

**Published:** 2022-09-27

**Authors:** Hideyuki Shimizu

**Affiliations:** Department of Cardiovascular Surgery, Keio University, Tokyo, Japan

I am honored to write this editorial for the launch of *General Thoracic and Cardiovascular Surgery Cases* (*GTCS Cases: GTCC*). On behalf of the editorial board of *General Thoracic and Cardiovascular Surgery* (*GTCS*), I, the Editor-in-Chief of *GTCS*, congratulate you on the launch of this new journal.

I would like to briefly introduce the history and current status of *GTCS*, which is the foundation of *GTCC*. The first issue of *GTCS* dates back to 1953, 5 years after the establishment of the Japanese Association for Thoracic Surgery (JATS). It became an English journal in 1998 and began to be disseminated around the world. The name of the journal was changed twice before the current name *GTCS* was adopted in 2007. To date, *GTCS* has played an important role as an academic journal of general thoracic, esophageal, and cardiovascular surgery in Japan and around the world. Currently, *GTCS* is the official journal of JATS and the Japanese Association for Chest Surgery (JACS) and is an affiliated journal of the Japanese Society for Cardiovascular Surgery (JSCVS). It acquired an impact factor (IF) of 1.219 for the first time in June 2018, which increased to 1.517 in 2020. Since attaining an IF, the number of submissions to *GTCS* from all around the world has increased dramatically. To further develop and nurture our future, we decided that *GTCS*, which previously dealt with a wide variety of categories, will shift its primary focus to the publication of original articles, including clinical and experimental studies, and that we would establish a new journal, *GTCC*, that would be dedicated to the publication of case reports.

As the field of medicine has developed through the accumulation of numerous individual experiences, excellent case reports are of great academic importance. Firstly, case reports are a treasure trove of information. Readers can often obtain detailed information that cannot be obtained from large studies or textbooks. By familiarizing themselves with cases that are encountered only rarely, readers can prepare for when they actually do encounter such cases. Tips and pitfalls that authors have learned provide many readers with a lot of knowledge and awareness, which ultimately results in improved quality of care. Secondly, being made aware of an author’s innovative ideas and thinking processes can be very instructive and may lead readers to come up with new ideas. From another perspective, writing and posting case reports are also a good training for future academic writing, and guidance from senior colleagues and peer reviewers can help authors to develop their skills as academic surgeons. Case reports have numerous other benefits in addition to contributing significantly to patients, medical progress, and increasing the IF of *GTCS*.

Our feelings about *GTCC* becoming independent of *GTCS* are comparable to the feelings of a parent on the eve of their baby’s birth. We sincerely hope that, with the cooperation of the editorial board, reviewers, and authors, *GTCC* will grow steadily and that it will come to play a major role in the field of academic surgery. Further, we strongly believe that the complementary roles of the two journals will contribute significantly to the development of thoracic and cardiovascular surgery.

We hope that many readers will be interested in *GTCC* and that they will submit many quality case reports to this exciting new journal.

